# Di-*tert*-Butyl 2,2′-[2,2′-methyl­enebis(naphthalene-2,1-diyldi­oxy)]diacetate

**DOI:** 10.1107/S1600536811003291

**Published:** 2011-01-29

**Authors:** Qamar Ali, Itrat Anis, M. Raza Shah, Seik Weng Ng

**Affiliations:** a H.E.J. Research Institute of Chemistry, International Center for Chemical and Biological Sciences, University of Karachi, Karachi 7527, Pakistan; b Department of Chemistry, University of Malaya, 50603 Kuala Lumpur, Malaysia

## Abstract

In the title compound, C_33_H_36_O_6_, two naphthalene ring systems are connected through a methyl­ene linkage [C—C—C = 114.9 (2)°]; the ring systems are aligned at an angle of 76.5 (1)°. Of the two –O–CH_2_–C(=O)–C(CH_3_)_3_ substituents, one adopts an extended conformation whereas the other is U-shaped. In the crystal, mol­ecules are linked *via* weak C—H⋯O hydrogen bonding, forming supra­molecular chains running along the *c* axis.

## Related literature

For two related structures, see: Ali *et al.* (2008 ▶); Mustafa *et al.* (2009 ▶).
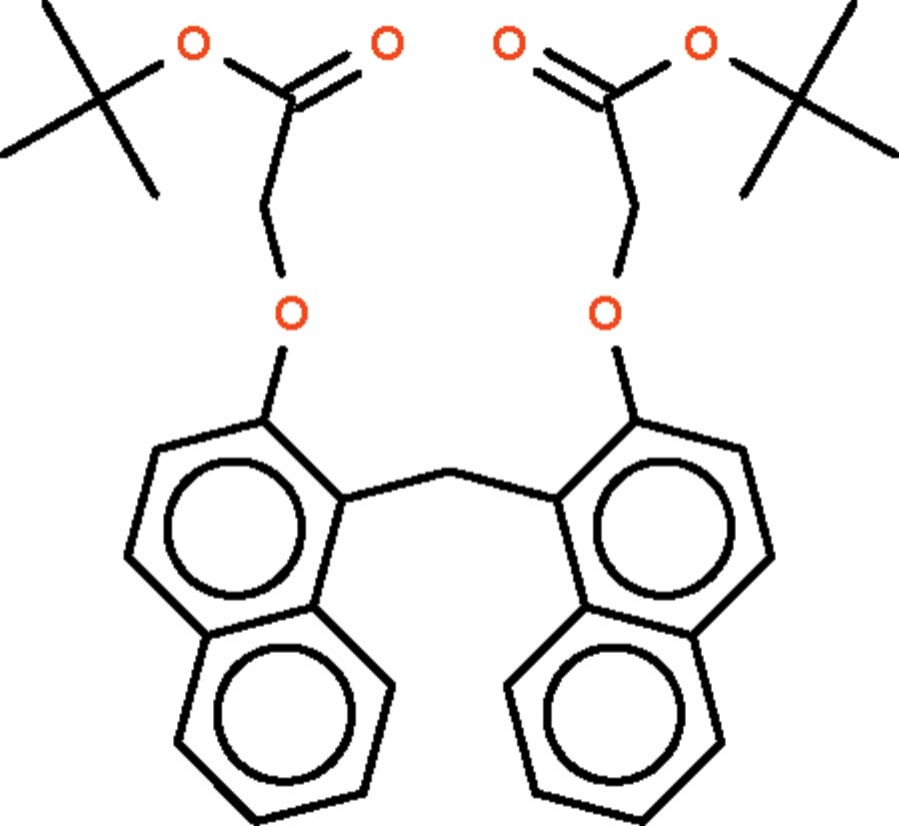


## Experimental

### 

#### Crystal data

C_33_H_36_O_6_
*M* *_r_* = 528.62
Triclinic, 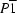

*a* = 8.9849 (5) Å
*b* = 11.8327 (6) Å
*c* = 13.7768 (6) Å
α = 79.804 (4)°
β = 74.115 (4)°
γ = 88.094 (4)°
*V* = 1386.33 (12) Å^3^
*Z* = 2
Mo *K*α radiation
μ = 0.09 mm^−1^
*T* = 100 K
0.35 × 0.15 × 0.05 mm

#### Data collection

Agilent SuperNova Dual diffractometer with an Atlas detector
Absorption correction: multi-scan (*CrysAlis PRO*; Agilent, 2010 ▶) *T*_min_ = 0.744, *T*_max_ = 1.000
11166 measured reflections
6119 independent reflections
3422 reflections with *I* > 2σ(*I*)
*R*_int_ = 0.055

#### Refinement

*R*[*F* ^2^ > 2σ(*F* ^2^)] = 0.070
*wR*(*F* ^2^) = 0.171
*S* = 1.03
6119 reflections
353 parameters
H-atom parameters constrained
Δρ_max_ = 0.32 e Å^−3^
Δρ_min_ = −0.24 e Å^−3^

### 

Data collection: *CrysAlis PRO* (Agilent, 2010 ▶); cell refinement: *CrysAlis PRO*; data reduction: *CrysAlis PRO*; program(s) used to solve structure: *SHELXS97* (Sheldrick, 2008 ▶); program(s) used to refine structure: *SHELXL97* (Sheldrick, 2008 ▶); molecular graphics: *X-SEED* (Barbour, 2001 ▶); software used to prepare material for publication: *publCIF* (Westrip, 2010 ▶).

## Supplementary Material

Crystal structure: contains datablocks global, I. DOI: 10.1107/S1600536811003291/xu5152sup1.cif

Structure factors: contains datablocks I. DOI: 10.1107/S1600536811003291/xu5152Isup2.hkl

Additional supplementary materials:  crystallographic information; 3D view; checkCIF report

## Figures and Tables

**Table 1:** Hydrogen-bond geometry (Å, °)

*D*—H⋯*A*	*D*—H	H⋯*A*	*D*⋯*A*	*D*—H⋯*A*
C4—H4*B*⋯O5^i^	0.98	2.46	3.419 (3)	166

Symmetry code: (i) 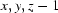.

**Fig. 1.**
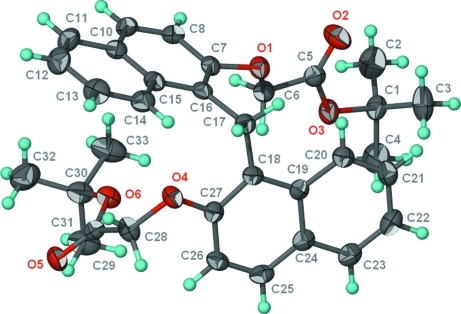
Thermal ellipsoid plot (Barbour, 2001) of C33H36O6 at the 70% probability level; hydrogen atoms are drawn as spheres of arbitrary radius.

## supplementary crystallographic information

### Comment

The crystal structure (Scheme I) continues from the studies on di-*tert*-butyl 2,2'-(biphenyl-2,2,'-diyldioxy)diacetate (Ali *et al.*, 2008) and di-*tert*-butyl (1,1'-binaphthyl-2,2'-dioxy)diacetate (Mustafa *et al.*, 2009). The title compound has two naphthyl ring systems connected through a methylene linkage [C–C–C 114.9 (2)°] (Fig. 1); the rings are aligned at a dihedral angle of 76.5 (1)°. In the crystal structure the molecules are linked via weak C—H···O hydrogen bonding to form one dimensional supra-molecular chains running along the c axis (Table 1).

### Experimental

1,1'-Methylenedi-2-naphthol (1 g, 3.3 mmol) was dissolved in acetone (25 ml). To the solution was added potassium cabonate (13.2 mmol) and *t*-butyl bromoacetate (3 ml, 19.8 mmol). The mixture was stirred at room temperature for 3 h. The solvent was evaporated under reduced pressure and the residue was dissolved in a mixture of water (50 ml) and dichloromethane (50 ml). The aqueous layer was extracted three times with dichloromethane. The combined organic phases were evaporated under reduced pressure and the solid material was recrystallized from *n*-hexane.

### Refinement

Carbon-bound H-atoms were placed in calculated positions [C—H 0.95 to 0.98 Å, *U*_iso_(H) 1.2 to 1.5*U*_eq_(C)] and were included in the refinement in the riding model approximation.

### Crystal data

**Table:**

C_33_H_36_O_6_	*Z* = 2
*M**_r_* = 528.62	*F*(000) = 564
Triclinic, *P*1	*D*_x_ = 1.266 Mg m^−^^3^
Hall symbol: -P 1	Mo *K*α radiation, λ = 0.71073 Å
*a* = 8.9849 (5) Å	Cell parameters from 2261 reflections
*b* = 11.8327 (6) Å	θ = 2.4–29.2°
*c* = 13.7768 (6) Å	µ = 0.09 mm^−^^1^
α = 79.804 (4)°	*T* = 100 K
β = 74.115 (4)°	Plate, colorless
γ = 88.094 (4)°	0.35 × 0.15 × 0.05 mm
*V* = 1386.33 (12) Å^3^	

### Data collection

**Table:**

Agilent SuperNova Dual diffractometer with an Atlas detector	6119 independent reflections
Radiation source: SuperNova (Mo) X-ray Source	3422 reflections with *I* > 2σ(*I*)
Mirror	*R*_int_ = 0.055
Detector resolution: 10.4041 pixels mm^-1^	θ_max_ = 27.5°, θ_min_ = 2.4°
ω scans	*h* = −11→11
Absorption correction: multi-scan (*CrysAlis PRO*; Agilent, 2010)	*k* = −14→15
*T*_min_ = 0.744, *T*_max_ = 1.000	*l* = −17→14
11166 measured reflections	

### Refinement

**Table:**

Refinement on *F*^2^	Secondary atom site location: difference Fourier map
Least-squares matrix: full	Hydrogen site location: inferred from neighbouring sites
*R*[*F*^2^ > 2σ(*F*^2^)] = 0.070	H-atom parameters constrained
*wR*(*F*^2^) = 0.171	*w* = 1/[σ^2^(*F*_o_^2^) + (0.0471*P*)^2^ + 0.6065*P*] where *P* = (*F*_o_^2^ + 2*F*_c_^2^)/3
*S* = 1.03	(Δ/σ)_max_ = 0.001
6119 reflections	Δρ_max_ = 0.32 e Å^−^^3^
353 parameters	Δρ_min_ = −0.24 e Å^−^^3^
0 restraints	Extinction correction: *SHELXL97* (Sheldrick, 2008), Fc^*^=kFc[1+0.001xFc^2^λ^3^/sin(2θ)]^-1/4^
Primary atom site location: structure-invariant direct methods	Extinction coefficient: 0.0041 (11)

### Fractional atomic coordinates and isotropic or equivalent isotropic displacement parameters (Å^2^)

**Table:**

	*x*	*y*	*z*	*U*_iso_*/*U*_eq_	
O1	0.5505 (2)	0.79128 (16)	0.41136 (14)	0.0244 (5)	
O2	0.5257 (2)	0.7736 (2)	0.22232 (16)	0.0394 (6)	
O3	0.7544 (2)	0.68264 (18)	0.19433 (14)	0.0296 (5)	
O4	0.8474 (2)	0.85959 (17)	0.63254 (14)	0.0250 (5)	
O5	0.9767 (2)	0.63445 (17)	0.79950 (15)	0.0295 (5)	
O6	0.8375 (2)	0.63524 (17)	0.68527 (15)	0.0264 (5)	
C1	0.7803 (3)	0.6858 (3)	0.0825 (2)	0.0284 (7)	
C2	0.6580 (4)	0.6145 (3)	0.0654 (3)	0.0440 (9)	
H2A	0.6573	0.5366	0.1042	0.066*	
H2B	0.6801	0.6112	−0.0078	0.066*	
H2C	0.5566	0.6490	0.0885	0.066*	
C3	0.7836 (5)	0.8078 (3)	0.0265 (2)	0.0435 (9)	
H3A	0.8621	0.8524	0.0415	0.065*	
H3B	0.6819	0.8420	0.0492	0.065*	
H3C	0.8091	0.8083	−0.0474	0.065*	
C4	0.9376 (4)	0.6325 (4)	0.0537 (2)	0.0496 (11)	
H4A	1.0139	0.6793	0.0684	0.074*	
H4B	0.9671	0.6287	−0.0196	0.074*	
H4C	0.9340	0.5548	0.0935	0.074*	
C5	0.6305 (3)	0.7267 (3)	0.2513 (2)	0.0245 (7)	
C6	0.6388 (3)	0.7062 (3)	0.3610 (2)	0.0242 (7)	
H6A	0.5969	0.6288	0.3959	0.029*	
H6B	0.7477	0.7108	0.3629	0.029*	
C7	0.5097 (3)	0.7688 (2)	0.5177 (2)	0.0209 (7)	
C8	0.4043 (3)	0.6790 (3)	0.5699 (2)	0.0241 (7)	
H8	0.3720	0.6284	0.5330	0.029*	
C9	0.3482 (3)	0.6644 (3)	0.6741 (2)	0.0252 (7)	
H9	0.2791	0.6022	0.7096	0.030*	
C10	0.3925 (3)	0.7412 (3)	0.7296 (2)	0.0238 (7)	
C11	0.3290 (3)	0.7285 (3)	0.8372 (2)	0.0311 (8)	
H11	0.2581	0.6673	0.8723	0.037*	
C12	0.3692 (4)	0.8038 (3)	0.8907 (2)	0.0339 (8)	
H12	0.3278	0.7938	0.9629	0.041*	
C13	0.4720 (4)	0.8961 (3)	0.8388 (2)	0.0332 (8)	
H13	0.4978	0.9492	0.8762	0.040*	
C14	0.5347 (3)	0.9099 (3)	0.7350 (2)	0.0276 (7)	
H14	0.6038	0.9727	0.7014	0.033*	
C15	0.4991 (3)	0.8324 (2)	0.6763 (2)	0.0228 (7)	
C16	0.5625 (3)	0.8441 (2)	0.5673 (2)	0.0204 (6)	
C17	0.6769 (3)	0.9388 (2)	0.5040 (2)	0.0226 (7)	
H17A	0.6793	0.9973	0.5468	0.027*	
H17B	0.6389	0.9764	0.4454	0.027*	
C18	0.8413 (3)	0.8990 (2)	0.4628 (2)	0.0207 (6)	
C19	0.9179 (3)	0.9095 (2)	0.3562 (2)	0.0207 (6)	
C20	0.8482 (3)	0.9589 (2)	0.2776 (2)	0.0245 (7)	
H20	0.7438	0.9829	0.2958	0.029*	
C21	0.9287 (3)	0.9723 (2)	0.1767 (2)	0.0277 (7)	
H21	0.8800	1.0068	0.1259	0.033*	
C22	1.0830 (3)	0.9357 (3)	0.1464 (2)	0.0299 (7)	
H22	1.1373	0.9447	0.0758	0.036*	
C23	1.1533 (3)	0.8874 (2)	0.2191 (2)	0.0271 (7)	
H23	1.2569	0.8622	0.1986	0.032*	
C24	1.0756 (3)	0.8740 (2)	0.3245 (2)	0.0234 (7)	
C25	1.1505 (3)	0.8281 (2)	0.3997 (2)	0.0245 (7)	
H25	1.2540	0.8026	0.3791	0.029*	
C26	1.0776 (3)	0.8193 (2)	0.5016 (2)	0.0232 (7)	
H26	1.1299	0.7883	0.5514	0.028*	
C27	0.9245 (3)	0.8564 (2)	0.5322 (2)	0.0215 (6)	
C28	0.9160 (4)	0.8127 (2)	0.7114 (2)	0.0270 (7)	
H28A	0.8618	0.8429	0.7741	0.032*	
H28B	1.0248	0.8402	0.6911	0.032*	
C29	0.9141 (3)	0.6837 (3)	0.7369 (2)	0.0232 (7)	
C30	0.8243 (3)	0.5085 (3)	0.6977 (2)	0.0292 (7)	
C31	0.9837 (4)	0.4598 (3)	0.6615 (3)	0.0387 (9)	
H31A	1.0309	0.4945	0.5899	0.058*	
H31B	0.9752	0.3765	0.6671	0.058*	
H31C	1.0484	0.4771	0.7041	0.058*	
C32	0.7430 (4)	0.4586 (3)	0.8080 (3)	0.0447 (9)	
H32A	0.6399	0.4917	0.8271	0.067*	
H32B	0.8034	0.4768	0.8528	0.067*	
H32C	0.7331	0.3751	0.8153	0.067*	
C33	0.7241 (4)	0.4949 (3)	0.6290 (3)	0.0429 (9)	
H33A	0.7777	0.5282	0.5581	0.064*	
H33B	0.6262	0.5344	0.6507	0.064*	
H33C	0.7033	0.4131	0.6334	0.064*	

### Atomic displacement parameters (Å^2^)

**Table:**

	*U*^11^	*U*^22^	*U*^33^	*U*^12^	*U*^13^	*U*^23^
O1	0.0270 (11)	0.0235 (12)	0.0217 (11)	0.0055 (9)	−0.0060 (9)	−0.0037 (9)
O2	0.0350 (13)	0.0560 (16)	0.0316 (13)	0.0176 (12)	−0.0144 (10)	−0.0135 (11)
O3	0.0316 (12)	0.0349 (13)	0.0189 (11)	0.0073 (10)	−0.0032 (9)	−0.0028 (9)
O4	0.0276 (11)	0.0252 (12)	0.0235 (11)	0.0058 (9)	−0.0099 (9)	−0.0043 (9)
O5	0.0336 (12)	0.0297 (13)	0.0261 (12)	0.0006 (10)	−0.0136 (10)	0.0014 (9)
O6	0.0282 (11)	0.0195 (11)	0.0346 (12)	0.0009 (9)	−0.0134 (9)	−0.0056 (9)
C1	0.0309 (17)	0.0292 (18)	0.0223 (16)	0.0011 (14)	−0.0033 (13)	−0.0032 (13)
C2	0.054 (2)	0.042 (2)	0.036 (2)	−0.0067 (18)	−0.0096 (17)	−0.0107 (17)
C3	0.071 (3)	0.036 (2)	0.0234 (18)	−0.0055 (19)	−0.0145 (17)	−0.0016 (15)
C4	0.045 (2)	0.074 (3)	0.0259 (19)	0.012 (2)	−0.0014 (16)	−0.0120 (18)
C5	0.0233 (16)	0.0220 (16)	0.0274 (16)	0.0005 (13)	−0.0061 (13)	−0.0034 (13)
C6	0.0243 (16)	0.0236 (17)	0.0237 (16)	0.0042 (13)	−0.0051 (12)	−0.0047 (13)
C7	0.0204 (15)	0.0214 (16)	0.0201 (15)	0.0079 (13)	−0.0053 (12)	−0.0030 (12)
C8	0.0192 (15)	0.0264 (17)	0.0272 (17)	0.0013 (13)	−0.0067 (13)	−0.0060 (13)
C9	0.0194 (15)	0.0238 (17)	0.0292 (17)	0.0009 (13)	−0.0044 (13)	−0.0002 (13)
C10	0.0193 (15)	0.0252 (17)	0.0259 (16)	0.0057 (13)	−0.0058 (12)	−0.0032 (13)
C11	0.0260 (16)	0.0321 (19)	0.0282 (17)	0.0019 (14)	0.0010 (13)	−0.0006 (14)
C12	0.0363 (19)	0.039 (2)	0.0234 (17)	0.0042 (16)	−0.0022 (14)	−0.0075 (15)
C13	0.0342 (18)	0.036 (2)	0.0311 (18)	0.0057 (16)	−0.0075 (15)	−0.0140 (15)
C14	0.0243 (16)	0.0266 (18)	0.0314 (17)	0.0037 (13)	−0.0063 (13)	−0.0067 (14)
C15	0.0218 (15)	0.0233 (17)	0.0245 (16)	0.0063 (13)	−0.0085 (12)	−0.0050 (13)
C16	0.0174 (14)	0.0195 (16)	0.0248 (16)	0.0052 (12)	−0.0076 (12)	−0.0033 (12)
C17	0.0222 (15)	0.0194 (16)	0.0262 (16)	0.0017 (12)	−0.0073 (13)	−0.0027 (12)
C18	0.0203 (15)	0.0131 (15)	0.0287 (16)	−0.0001 (12)	−0.0082 (12)	−0.0015 (12)
C19	0.0225 (15)	0.0135 (15)	0.0256 (16)	−0.0043 (12)	−0.0070 (12)	−0.0002 (12)
C20	0.0243 (15)	0.0196 (16)	0.0298 (17)	−0.0003 (13)	−0.0091 (13)	−0.0024 (13)
C21	0.0350 (18)	0.0221 (17)	0.0261 (17)	−0.0008 (14)	−0.0112 (14)	0.0001 (13)
C22	0.0329 (18)	0.0251 (18)	0.0268 (17)	−0.0037 (14)	0.0005 (14)	−0.0049 (13)
C23	0.0264 (16)	0.0178 (16)	0.0339 (18)	−0.0011 (13)	−0.0039 (14)	−0.0033 (13)
C24	0.0234 (16)	0.0157 (15)	0.0296 (17)	−0.0014 (12)	−0.0048 (13)	−0.0036 (12)
C25	0.0185 (15)	0.0174 (16)	0.0369 (18)	0.0018 (12)	−0.0059 (13)	−0.0061 (13)
C26	0.0226 (15)	0.0159 (15)	0.0336 (17)	0.0006 (12)	−0.0133 (13)	−0.0020 (13)
C27	0.0236 (15)	0.0160 (15)	0.0244 (16)	−0.0013 (12)	−0.0067 (12)	−0.0015 (12)
C28	0.0356 (17)	0.0216 (17)	0.0274 (17)	0.0017 (14)	−0.0144 (14)	−0.0053 (13)
C29	0.0202 (15)	0.0253 (17)	0.0233 (16)	−0.0016 (13)	−0.0037 (13)	−0.0052 (13)
C30	0.0271 (17)	0.0214 (17)	0.0418 (19)	0.0024 (13)	−0.0131 (15)	−0.0072 (14)
C31	0.0353 (19)	0.033 (2)	0.055 (2)	0.0065 (16)	−0.0183 (17)	−0.0177 (17)
C32	0.039 (2)	0.039 (2)	0.054 (2)	−0.0120 (17)	−0.0097 (17)	−0.0037 (18)
C33	0.039 (2)	0.032 (2)	0.069 (3)	0.0048 (16)	−0.0276 (19)	−0.0207 (19)

### Geometric parameters (Å, °)

**Table:**

O1—C7	1.388 (3)	C14—C15	1.422 (4)
O1—C6	1.420 (3)	C14—H14	0.9500
O2—C5	1.203 (3)	C15—C16	1.436 (4)
O3—C5	1.324 (3)	C16—C17	1.521 (4)
O3—C1	1.488 (3)	C17—C18	1.521 (4)
O4—C27	1.373 (3)	C17—H17A	0.9900
O4—C28	1.413 (3)	C17—H17B	0.9900
O5—C29	1.210 (3)	C18—C27	1.386 (4)
O6—C29	1.322 (3)	C18—C19	1.426 (4)
O6—C30	1.484 (4)	C19—C20	1.425 (4)
C1—C2	1.500 (5)	C19—C24	1.435 (4)
C1—C4	1.508 (4)	C20—C21	1.364 (4)
C1—C3	1.509 (4)	C20—H20	0.9500
C2—H2A	0.9800	C21—C22	1.411 (4)
C2—H2B	0.9800	C21—H21	0.9500
C2—H2C	0.9800	C22—C23	1.360 (4)
C3—H3A	0.9800	C22—H22	0.9500
C3—H3B	0.9800	C23—C24	1.410 (4)
C3—H3C	0.9800	C23—H23	0.9500
C4—H4A	0.9800	C24—C25	1.407 (4)
C4—H4B	0.9800	C25—C26	1.364 (4)
C4—H4C	0.9800	C25—H25	0.9500
C5—C6	1.510 (4)	C26—C27	1.404 (4)
C6—H6A	0.9900	C26—H26	0.9500
C6—H6B	0.9900	C28—C29	1.504 (4)
C7—C16	1.380 (4)	C28—H28A	0.9900
C7—C8	1.403 (4)	C28—H28B	0.9900
C8—C9	1.365 (4)	C30—C33	1.504 (4)
C8—H8	0.9500	C30—C31	1.513 (4)
C9—C10	1.416 (4)	C30—C32	1.514 (5)
C9—H9	0.9500	C31—H31A	0.9800
C10—C11	1.418 (4)	C31—H31B	0.9800
C10—C15	1.423 (4)	C31—H31C	0.9800
C11—C12	1.366 (4)	C32—H32A	0.9800
C11—H11	0.9500	C32—H32B	0.9800
C12—C13	1.410 (5)	C32—H32C	0.9800
C12—H12	0.9500	C33—H33A	0.9800
C13—C14	1.368 (4)	C33—H33B	0.9800
C13—H13	0.9500	C33—H33C	0.9800
			
C7—O1—C6	116.39 (19)	C16—C17—H17A	108.5
C5—O3—C1	122.7 (2)	C18—C17—H17A	108.5
C27—O4—C28	119.9 (2)	C16—C17—H17B	108.5
C29—O6—C30	121.3 (2)	C18—C17—H17B	108.5
O3—C1—C2	109.5 (3)	H17A—C17—H17B	107.5
O3—C1—C4	101.9 (2)	C27—C18—C19	118.0 (2)
C2—C1—C4	111.5 (3)	C27—C18—C17	118.4 (2)
O3—C1—C3	110.8 (2)	C19—C18—C17	123.5 (2)
C2—C1—C3	111.8 (3)	C20—C19—C18	123.2 (2)
C4—C1—C3	110.9 (3)	C20—C19—C24	117.1 (3)
C1—C2—H2A	109.5	C18—C19—C24	119.6 (2)
C1—C2—H2B	109.5	C21—C20—C19	121.2 (3)
H2A—C2—H2B	109.5	C21—C20—H20	119.4
C1—C2—H2C	109.5	C19—C20—H20	119.4
H2A—C2—H2C	109.5	C20—C21—C22	121.1 (3)
H2B—C2—H2C	109.5	C20—C21—H21	119.4
C1—C3—H3A	109.5	C22—C21—H21	119.4
C1—C3—H3B	109.5	C23—C22—C21	119.4 (3)
H3A—C3—H3B	109.5	C23—C22—H22	120.3
C1—C3—H3C	109.5	C21—C22—H22	120.3
H3A—C3—H3C	109.5	C22—C23—C24	121.4 (3)
H3B—C3—H3C	109.5	C22—C23—H23	119.3
C1—C4—H4A	109.5	C24—C23—H23	119.3
C1—C4—H4B	109.5	C25—C24—C23	121.3 (3)
H4A—C4—H4B	109.5	C25—C24—C19	119.0 (3)
C1—C4—H4C	109.5	C23—C24—C19	119.7 (2)
H4A—C4—H4C	109.5	C26—C25—C24	121.3 (2)
H4B—C4—H4C	109.5	C26—C25—H25	119.3
O2—C5—O3	126.5 (3)	C24—C25—H25	119.3
O2—C5—C6	124.6 (3)	C25—C26—C27	119.4 (2)
O3—C5—C6	108.9 (2)	C25—C26—H26	120.3
O1—C6—C5	108.5 (2)	C27—C26—H26	120.3
O1—C6—H6A	110.0	O4—C27—C18	114.3 (2)
C5—C6—H6A	110.0	O4—C27—C26	123.0 (2)
O1—C6—H6B	110.0	C18—C27—C26	122.6 (3)
C5—C6—H6B	110.0	O4—C28—C29	115.5 (2)
H6A—C6—H6B	108.4	O4—C28—H28A	108.4
C16—C7—O1	117.9 (3)	C29—C28—H28A	108.4
C16—C7—C8	122.8 (3)	O4—C28—H28B	108.4
O1—C7—C8	119.0 (3)	C29—C28—H28B	108.4
C9—C8—C7	119.8 (3)	H28A—C28—H28B	107.5
C9—C8—H8	120.1	O5—C29—O6	126.4 (3)
C7—C8—H8	120.1	O5—C29—C28	120.9 (3)
C8—C9—C10	120.4 (3)	O6—C29—C28	112.6 (2)
C8—C9—H9	119.8	O6—C30—C33	102.1 (2)
C10—C9—H9	119.8	O6—C30—C31	109.2 (3)
C9—C10—C11	120.2 (3)	C33—C30—C31	112.0 (3)
C9—C10—C15	119.5 (3)	O6—C30—C32	110.0 (2)
C11—C10—C15	120.2 (3)	C33—C30—C32	110.4 (3)
C12—C11—C10	120.4 (3)	C31—C30—C32	112.7 (3)
C12—C11—H11	119.8	C30—C31—H31A	109.5
C10—C11—H11	119.8	C30—C31—H31B	109.5
C11—C12—C13	120.1 (3)	H31A—C31—H31B	109.5
C11—C12—H12	119.9	C30—C31—H31C	109.5
C13—C12—H12	119.9	H31A—C31—H31C	109.5
C14—C13—C12	120.5 (3)	H31B—C31—H31C	109.5
C14—C13—H13	119.8	C30—C32—H32A	109.5
C12—C13—H13	119.8	C30—C32—H32B	109.5
C13—C14—C15	121.5 (3)	H32A—C32—H32B	109.5
C13—C14—H14	119.2	C30—C32—H32C	109.5
C15—C14—H14	119.2	H32A—C32—H32C	109.5
C14—C15—C10	117.3 (3)	H32B—C32—H32C	109.5
C14—C15—C16	123.1 (3)	C30—C33—H33A	109.5
C10—C15—C16	119.6 (3)	C30—C33—H33B	109.5
C7—C16—C15	117.7 (3)	H33A—C33—H33B	109.5
C7—C16—C17	118.9 (2)	C30—C33—H33C	109.5
C15—C16—C17	123.3 (3)	H33A—C33—H33C	109.5
C16—C17—C18	114.9 (2)	H33B—C33—H33C	109.5
			
C5—O3—C1—C2	−65.1 (3)	C16—C17—C18—C27	−66.1 (3)
C5—O3—C1—C4	176.7 (3)	C16—C17—C18—C19	117.8 (3)
C5—O3—C1—C3	58.7 (4)	C27—C18—C19—C20	−175.8 (3)
C1—O3—C5—O2	0.0 (5)	C17—C18—C19—C20	0.3 (4)
C1—O3—C5—C6	178.6 (3)	C27—C18—C19—C24	1.3 (4)
C7—O1—C6—C5	163.0 (2)	C17—C18—C19—C24	177.3 (3)
O2—C5—C6—O1	−26.4 (4)	C18—C19—C20—C21	176.7 (3)
O3—C5—C6—O1	155.0 (2)	C24—C19—C20—C21	−0.3 (4)
C6—O1—C7—C16	119.0 (3)	C19—C20—C21—C22	1.2 (5)
C6—O1—C7—C8	−67.8 (3)	C20—C21—C22—C23	−0.8 (5)
C16—C7—C8—C9	0.9 (4)	C21—C22—C23—C24	−0.6 (5)
O1—C7—C8—C9	−172.0 (2)	C22—C23—C24—C25	−177.6 (3)
C7—C8—C9—C10	1.7 (4)	C22—C23—C24—C19	1.4 (4)
C8—C9—C10—C11	177.4 (2)	C20—C19—C24—C25	178.1 (3)
C8—C9—C10—C15	−1.5 (4)	C18—C19—C24—C25	0.9 (4)
C9—C10—C11—C12	−178.7 (3)	C20—C19—C24—C23	−0.9 (4)
C15—C10—C11—C12	0.1 (4)	C18—C19—C24—C23	−178.1 (3)
C10—C11—C12—C13	1.3 (4)	C23—C24—C25—C26	177.4 (3)
C11—C12—C13—C14	−1.3 (4)	C19—C24—C25—C26	−1.6 (4)
C12—C13—C14—C15	0.0 (4)	C24—C25—C26—C27	0.2 (4)
C13—C14—C15—C10	1.4 (4)	C28—O4—C27—C18	176.4 (3)
C13—C14—C15—C16	179.9 (2)	C28—O4—C27—C26	−6.9 (4)
C9—C10—C15—C14	177.5 (2)	C19—C18—C27—O4	173.9 (2)
C11—C10—C15—C14	−1.4 (4)	C17—C18—C27—O4	−2.3 (4)
C9—C10—C15—C16	−1.1 (4)	C19—C18—C27—C26	−2.8 (4)
C11—C10—C15—C16	180.0 (2)	C17—C18—C27—C26	−179.0 (3)
O1—C7—C16—C15	169.5 (2)	C25—C26—C27—O4	−174.3 (3)
C8—C7—C16—C15	−3.5 (4)	C25—C26—C27—C18	2.1 (4)
O1—C7—C16—C17	−7.1 (3)	C27—O4—C28—C29	−75.3 (3)
C8—C7—C16—C17	179.9 (2)	C30—O6—C29—O5	−2.0 (4)
C14—C15—C16—C7	−175.0 (2)	C30—O6—C29—C28	178.8 (2)
C10—C15—C16—C7	3.5 (3)	O4—C28—C29—O5	176.2 (2)
C14—C15—C16—C17	1.5 (4)	O4—C28—C29—O6	−4.5 (4)
C10—C15—C16—C17	180.0 (2)	C29—O6—C30—C33	177.8 (2)
C7—C16—C17—C18	−74.6 (3)	C29—O6—C30—C31	−63.6 (3)
C15—C16—C17—C18	109.0 (3)	C29—O6—C30—C32	60.6 (3)

### Hydrogen-bond geometry (Å, °)

**Table:**

*D*—H···*A*	*D*—H	H···*A*	*D*···*A*	*D*—H···*A*
C4—H4B···O5^i^	0.98	2.46	3.419 (3)	166

Symmetry codes: (i) *x*, *y*, *z*−1.
